# A BRAF V600E Mutation in RET-Negative Medullary Thyroid Cancer

**DOI:** 10.1155/2020/7641940

**Published:** 2020-03-10

**Authors:** Richard J. Robbins, Jessica S. Thomas, Patricia Mejia Osuna, Jawairia Shakil

**Affiliations:** ^1^Weill Cornell Medicine, New York, NY, USA; ^2^Department of Pathology and Genomic Medicine, Houston Methodist Hospital, Houston, TX, USA; ^3^Division of Endocrinology, Diabetes, and Metabolism, Department of Medicine, Houston Methodist Hospital, Houston, TX, USA

## Abstract

We report the case of a woman with a sporadic medullary thyroid carcinoma. Genomic analysis found that her tumor did not contain any common *RET* mutations but did harbor a *BRAF* V600E mutation. Only one other well-confirmed example of the BRAF V600E mutation has been reported in an MTC patient. We conclude that this common *BRAF* mutation may independently drive neoplastic transformation of human parafollicular C cells.

## 1. Introduction

Medullary thyroid cancer (MTC) accounts for 1–2% of all new thyroid cancers in the US [1]. Approximately, 20% of MTC presents with a hereditary pattern, usually caused by germline mutations in the *RET* gene. The remaining MTCs are nonfamilial, and 50% of these have a somatic *RET* mutation. Patients with somatic *RET* mutations (most commonly p.M918T) have shorter overall survival compared with MTC without a *RET* mutation [2]. Less commonly sporadic MTC does not harbor a *RET* mutation, but is associated with *RAS* or *ALK* gene rearrangements. In general, *BRAF* point mutations have been found to be absent in non-*RET*-mutated MTC specimens [3].

## 2. Case Presentation

**Figure 1:**
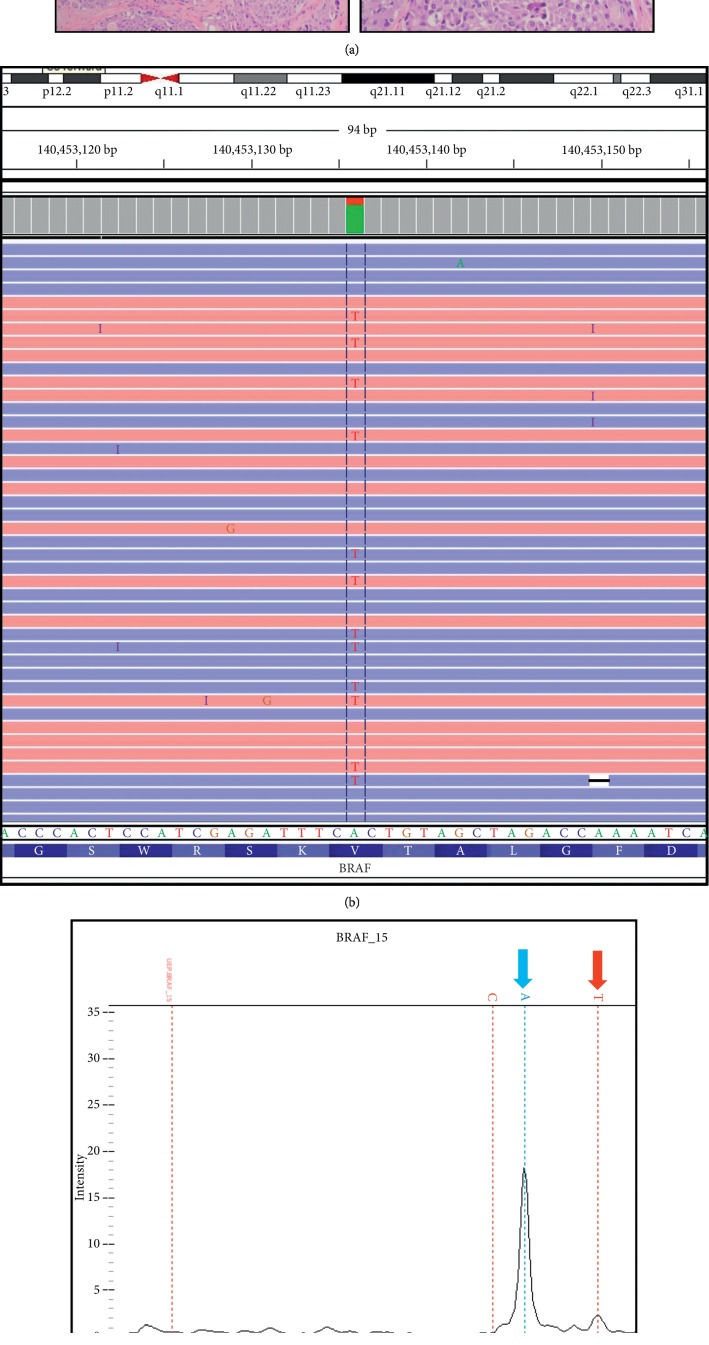
(a) An H&E-stained tissue section of the thyroid tumor at 100x (left) and 200x (right) magnification is shown. The tissue shows solid sheets and nests of cells separated by hyalinized fibrovascular stroma. Interspersed between the cells is a homogeneous, acellular, eosinophilic, extracellular matrix material, consistent with amyloid (solid yellow arrow). There are numerous scattered calcifications (solid blue arrow). Cytologically, the tumor cells are spindled, plasmacytoid, or polyhedral in shape with round to oval nuclei showing stippled, fine, uniform salt and pepper nuclear chromatin, and a moderate amount of pink granular cytoplasm. These findings are consistent with the diagnosis of medullary thyroid carcinoma. (b) Integrated Genomics Viewer representative display of the mutated sequence in *BRAF* detected by the next-generation sequencing analysis. Separate sequencing reads at this nucleotide position (1799) are shown. A (green) to T (red) nucleotide substitution (variant allele frequency, 10.3%) results in an amino acid substitution (glutamic acid for valine) at codon 600 of *BRAF*. (c) Presence of the *BRAF* p.V600E mutation was confirmed by multiplexed PCR and single-base extension with mass spectrometry analysis on the Sequenom instrument (Agena Biosciences). The mass (*x*-axis) and intensity (*y*-axis) of the *BRAF* peaks are shown. The blue arrow indicates the wildtype *BRAF* peak, and the red arrow indicates the mutant *BRAF* peak. The T to A nucleotide substitution shows results in an amino acid substitution (glutamic acid for valine) at codon 600 of *BRAF*.

We report a 66 y/o African American female who was found incidentally to have a 1.3 cm thyroid nodule. A fine needle aspiration biopsy demonstrated cytological features of MTC with positive staining for calcitonin (CT). There was no family history of thyroid cancer or any other endocrine neoplasia. Serum CT was elevated at 75 pmol/L, and the serum CEA was within normal limits. Blood DNA analysis was negative for *RET* germline mutations in exons 5, 8, 10, 11, and 13–16. Pathological analysis after a total thyroidectomy and central neck lymph node dissection revealed a 1.8 cm MTC (Figure 1(a)) with multifocality, extrathyroidal extension, and MTC in 6 out of 10 lymph nodes. The tumor was microdissected out and analyzed for 50 of the most common oncogene mutations by next-generation sequencing, including *RET* and *RAS* genes. The only abnormality detected was a *BRAF* p.V600E mutation (Figure 1(b)). The presence of a *BRAF* p.V600E mutation was then confirmed by an alternate testing platform (Figure 1(c)). The *BRAF* p.V600E was detected in this patient's tumor at a low allele frequency (5–10%) on both testing platforms, likely reflecting the tumor heterogeneity and low tumor burden of the tissue sample. Imaging studies with neck and chest CT scan and MRI of the liver were negative for metastatic disease. One month following thyroidectomy, her serum calcitonin was 70.1 pmol/L. At a 2 year follow-up, she remained asymptomatic with a serum CT level of 26.3 pmol/L, a CEA of 2 mcg/L, and no clinical evidence of disease on exam or by imaging.

## 3. Materials and Methods

On standard pathologic assessment, the tumor tissue demonstrated morphologic features consistent with a diagnosis of medullary thyroid carcinoma (Figure 1(a)). There was no histological evidence of a papillary thyroid cancer (PTC) in any other sections of the thyroid specimen, including no admixture of PTC within the MTC. Furthermore, none of the resected lymph nodes contained any histological evidence of PTC. Somatic mutation analysis of the tumor tissue was performed using a 50 gene next-generation sequencing assay (Ion AmpliSeq Tumor Hotspot v2, Life Technologies) and single nucleotide variant genotyping by single-base extension followed by mass spectroscopy analysis (Oncocarta Panel and Sequenom, Agena Biosciences). The report of this case was approved by the Houston Methodist Institutional Review Board (IRB #1014–0216).

## 4. Discussion

The vast majority of studies analyzing somatic mutations in sporadic cases of MTC continue to demonstrate that mutations other than *RET* and *RAS* are still very rare or absent. Our patient had histologically classical MTC, without concurrent PTC, but unexpectedly had a *BRAF* p.V600E mutation which is typically only associated with PTC. Only one other report has confirmed the presence of the V600E mutation in one MTC patient in Korea [4]. One remarkable report from Greece found that 68% of their MTC cases had a *BRAF* p.V600E mutation [5]. This extremely atypical result has not been confirmed and certain methodological details may have led to false positive results [4]. However, numerous other investigators [6–8] have not found any *BRAF* point mutations in hundreds of *RET* and non-*RET*-mutated MTC cases. One MTC case was found to have a unique fusion between *PARP12* (exons 1–9) and *BRAF* (exons 11–18), creating a putative oncogene [9]. B-Raf is a serine/threonine-protein kinase which is the key element in the RET/Raf/MEK/ERK pathway that is often activated in MTC. Our rare finding suggests that a gain of function *BRAF* point mutation may be able to drive malignant transformation of human parafollicular C cells, through the MEK/ERK pathway in the absence of a *RET* mutation. However, the possibility exists that this *BRAF* point mutation, which was present at a low allelic frequency, might not be the causative or “driver” mutation in this case.
